# Characterization and Discrimination of Pure Standards of Phenolic Compounds Using FTIR Spectroscopy in the Terahertz Range

**DOI:** 10.3390/foods14213737

**Published:** 2025-10-31

**Authors:** Audrey Pissard, Vincent Baeten, Quentin Arnould, Hervé Rogez, François Stevens

**Affiliations:** 1 Walloon Agricultural Research Centre (CRA-W), Knowledge and Valorization of Agricultural Products Department, Chaussée de Namur, 24, 5030 Gembloux, Belgium; v.baeten@cra.wallonie.be (V.B.); q.arnould@cra.wallonie.be (Q.A.); f.stevens@cra.wallonie.be (F.S.); 2 Centre for Valorisation of Amazonian Bioactive Compounds (CVACBA), Institute of Biological Sciences, Federal University of Para, Belém-Pará 66075-110, Brazil; herverogez@gmail.com

**Keywords:** Fourier transform infrared spectroscopy, FTIR, far infrared, terahertz, THz, attenuated total reflectance, ATR, phenolic compounds, phenolic acids, flavonoids

## Abstract

Phenolic compounds (PCs) are bioactive molecules synthesized by plants and recognized for their antioxidant, antimicrobial, and anti-inflammatory properties. Traditional methods for their analysis, such as HPLC or GC, are time-consuming and costly, which motivates the exploration of faster and non-destructive alternatives. This study investigates the potential of Fourier Transform Infrared (FTIR) spectroscopy in the Terahertz (THz) range for the identification and discrimination of PCs. Fifty-five pure standards, including phenolic acids and flavonoids, were analyzed using an FTIR spectrometer equipped with an Attenuated Total Reflectance (ATR) accessory. Measurements were performed at room temperature with 2–4 replicates. Repeatability and time reproducibility were good overall but decreased towards lower frequencies. Partial Least Squares Discriminant Analysis (PLS-DA) was applied as an exploratory tool to assess the global spectral variability among PCs and to determine whether their class or family was associated with systematic spectral features. The models achieved moderate to high accuracy in distinguishing between phenolic acids, flavonoids, and their subclasses. This study demonstrates the ability of THz spectroscopy to discriminate pure phenolic compounds despite their complex spectral profiles and represents a first step toward its application in real food products. Future work should address the limited sensitivity of FTIR spectroscopy for trace detection and the high absorption of water in the FIR–THz range, through experiments on dry mixtures of pure PCs and model food supplements to establish suitable conditions for food analysis.

## 1. Introduction

Phenolic compounds (PCs) are the most abundant group of secondary metabolites in plants and are widely distributed across species. They play crucial roles in plant physiology, contributing to quality traits such as color, flavor, and stress resistance, and are key components of plant defense mechanisms. In addition, PCs display a broad spectrum of biological activities, including antioxidant, antimicrobial, anti-inflammatory, and anticancer effects, and are associated with the prevention of cardiovascular and metabolic disorders as well as diseases linked to oxidative stress [1,2]. Structurally, PCs share a common aromatic backbone with one or more hydroxyl substituents and can be divided into several major classes, including phenolic acids, flavonoids, tannins, lignans, and stilbenes. Their molecular weights range from simple phenolic acids (94–170 Da) to complex tannins (500–20,000 Da), and more than 10,000 distinct structures have been reported to date.

Different reviews have summarized the occurrence, extraction, and analysis of PCs, with a strong emphasis on their biological activities [2,3]. Conventional analytical techniques such as high-performance liquid chromatography (HPLC), gas chromatography (GC), and gas chromatography–mass spectrometry (GC–MS) have been extensively employed. While accurate, these methods are costly, labor-intensive, and require large amounts of solvents and reagents. As alternatives, vibrational spectroscopic techniques such as near-infrared (NIR), mid-infrared (MIR), and Raman spectroscopy offer many advantages over conventional chemical analysis methods and have been widely applied for the detection of biological molecules [4]. Rapid, non-destructive screening approaches based on spectroscopy are of particular interest for identifying and discriminating PCs. Johnson et al. [5] reviewed recent literature on the use of infrared spectroscopy (NIR and MIR) for the quantification of bioactive compounds in food products, highlighting the growing number of studies applying these techniques to PCs in different food matrices. Many of these investigations target the total polyphenol content or the quantification of specific PCs within the matrix. Abbas et al. [6], for instance, analyzed pure phenolic standards (powders) with a MIR-FTIR spectrometer in order to identify their main spectral bands and to evaluate the potential to discriminate between PCs or families of PCs.

The part of the infrared with the larger wavelengths, at the interface of the microwave region, is named the far infrared (FIR) region (10 to 400 cm^−1^; approx. 0.3 to 12 THz) or the Terahertz (THz) region (3.3 cm^−1^ to 333 cm^−1^; approx. 0.1 to 10 THz). As noted in a recent review [7], the absorption bands in this region arise mainly from intermolecular, lattice, and skeletal vibrations, and may also involve rotational transitions. The main techniques of spectroscopy in this spectral region, globally known as THz spectroscopy, are THz time-domain spectroscopy (THz-TDS) and FIR Fourier-transform spectroscopy (FTIR). According to [8], THz-TDS provides a better signal-to-noise ratio (SNR) below 3 THz (approx. 100 cm^−1^), whereas FIR-FTIR performs better above 5 THz (approx. 167 cm^−1^).

THz spectroscopy is a relatively recent technique, offering rapid, non-destructive, and non-ionizing analysis. It has been increasingly applied to food quality and safety testing [9,10,11]. However, only a few studies have explored its use for characterizing phenolic compounds (PCs). Some flavonoids have been shown to exhibit well-defined bands in the THz region, supporting the feasibility of this method for their identification and quantification [12,13]. More recently, the biomolecular properties of ten common PCs were investigated using THz-TDS in the 0.2–2.5 THz (approx. 7–83 cm^−1^) range [4]. These compounds displayed distinct absorption bands, enabling both qualitative identification and quantitative analysis.

Our study builds upon previous research on the characterization of phenolic compounds (PCs) using vibrational spectroscopy techniques such as Raman and mid-infrared (MIR) spectroscopy [6,14]. The aim was to investigate their spectral characteristics in a relatively under-investigated spectral region. Spectral features of PC standards, including phenolic acids and flavonoids, were examined using FTIR spectroscopy between 0.90 and 20.35 THz (approx. 30–679 cm^−1^), thus encompassing both the THz and far-infrared (FIR) ranges. The repeatability of the spectra was evaluated through successive measurements, while time-based reproducibility was assessed by monitoring spectral stability over extended periods. A spectral database was constructed, and the feasibility of discriminating between different PC families or classes was explored. This approach based on pure standards represents a first step towards more practical applications aiming at characterizing real food products. These latter would certainly imply effects from interactions between PCs and food matrices, and water in particular. Here, we propose a more fundamental exploration of the interaction between PCs and radiation in the THz range. 

## 2. Materials and Methods

### 2.1. Standards

A total of 55 standards were analyzed (Table 1), representing the two main families of PCs: 28 phenolic acids and 27 flavonoids. These were sourced from three different suppliers (listed in Table 1), with some compounds represented by standards from multiple suppliers. Altogether, the dataset comprised 39 distinct PCs, including 26 flavonoids and 13 phenolic acids. Prior to analysis, all standards were stored under constant temperature in a chamber without any additional preparation step. Measurements were performed at room temperature with 2 to 4 replicates. Cyanidin chloride was measured only once due to its limited availability. In total, 141 spectra were obtained.

### 2.2. Experimental Setup

The samples were analyzed in powder form. Spectra were collected using a Attenuated Total Reflectance (ATR) Platinum accessory integrated into a Vertex 70 spectrometer (Bruker Optics, Ettlingen, Germany), equipped with an EKOM DK50PLUS/M compressor to maintain the instrument under low humidity and CO_2_ conditions. The ATR module, fitted with an integrated press, ensured optimal contact between the sample and the crystal. Approximately 10 mg of material were placed on the ATR crystal for each measurement. Each spectrum represented the average of 128 scans, recorded in the absorbance range of 29.98–678.92 cm^−1^ (0.90–20.35 THz), with 674 signals equally distributed over this range. A background spectrum (ambient air) was acquired before each sample measurement. Spectral acquisition was performed with OPUS software (version 6.5).

### 2.3. Spectral Pre-Processing

Infrared spectra acquisition often includes irrelevant information caused by physical effects such as variations in optical path length and scattering. These cause additive effects, such as vertical shifts in the entire spectrum, and multiplicative effects, such as variations in absorption band intensities. These features reflect physical rather than chemical properties and can significantly affect statistical analyses and predictive model performance. Therefore, mathematical pre-processing is necessary to correct these artifacts. Derivatives help reduce additive effects and emphasize absorption bands, but they also amplify noise, reducing the SNR. The Savitzky–Golay method smooths these derivatives by fitting a local polynomial to subsets of the data before differentiation, providing a more stable estimate. In this study, the Savitzky–Golay first derivative with a second-order polynomial and a window of seven points was used. First results showed that the method effectively corrected baseline offset without adding noise. The pre-processing workflow was applied with all statistical analyses and predictive modeling applications presented here. However, the discussion on spectral features was based on raw spectra, as derivative pre-processing can modify the shape of the absorption bands and shift their position.

### 2.4. Assessment of Repeatability and Time-Reproducibility

To evaluate the potential of THz spectroscopy for characterizing PCs, the consistency of spectra was assessed in terms of repeatability (successive measurements of the same sample) and time-reproducibility (measurements of the same sample spaced over time). For repeatability, all samples listed in Table 1 were used. For time-reproducibility, a subset of four PCs representing different classes was selected (Table 2), chosen based on sample availability to allow repeated measurements. Each of these four PCs was measured five times at weekly intervals. At each session, duplicate measurements were taken and averaged.

For the repeatability and reproducibility assessment, the same frequency-wise indicator of class consistency was used, namely the Relative Standard Deviation (RSD). For a given class *k*, it is defined as

$${RSD}_{k}\left(\upsilon \right)=\frac{\sigma_{k}^{inter}\left(\upsilon \right)}{\sum_{i=1}^{n_{k}}\sigma_{i}^{intra}/n_{k}}$$

with

$$\sigma_{k}^{inter}\left(\upsilon \right)=\frac{\sum_{i=1}^{n_{k}}{\left(A_{k}^{i}\left(\upsilon \right)-\frac{\sum_{j=1}^{n_{k}}A_{k}^{j}\left(\upsilon \right)}{n_{k}}\right)}^{2}}{n_{k}-1},$$

the standard deviation over spectra of class k at the frequency υ, $A_{k}^{i}\left(\upsilon \right)$ the absorbance of the ith spectrum of class k at the frequency υ, $n_{k}$ the number of spectra of class k and $\sigma_{i}^{intra}$ the standard deviation of absorbance values within the ith spectrum.

Regarding repeatability and time-reproducibility, the key difference lies in the class definition. For repeatability, two spectra belong to the same class if they correspond to successive measurements of the same sample without any sample manipulation between measurements. For reproducibility, two spectra belong to the same class if they represent measurements of the same standard, possibly at different times.

A value of ${RSD}_{k}\left(\upsilon \right)=1$ indicates that between-measurement variability equals the average variability within a single spectrum. Hence, good repeatability or reproducibility at frequency υ implies ${RSD}_{k}\left(\upsilon \right)\ll 1$. The mean RSD over all classes (*k*)—i.e., 54 classes for repeatability and 4 for reproducibility—provides a general estimate of the repeatability or reproducibility of PCs at different frequencies for our instrument and measurement settings.

### 2.5. Predictive Classification Modeling

In this study, partial least squares discriminant analysis (PLS-DA) was used as an exploratory tool to globally assess the variability among phenolic compounds (PCs) and to investigate whether belonging to a given class or family was associated with systematic spectral features. Compared with PCA, PLS-DA provides an objective and quantifiable discrimination outcome, avoiding the subjective interpretation that can arise from visual assessment of PCA score plots. Moreover, PLS-DA loadings are directly related to class discrimination and therefore highlight the specific spectral features contributing to group separation.

In practice, three case studies were considered: (CS1) discrimination between the two main families of PCs (flavonoids vs. phenolic acids); (CS2) the discrimination among flavonoids between the flavonols (the most represented class) and the other classes; (CS3) discrimination among phenolic acids between the hydroxycinnamic acids and the hydroxybenzoic acids. For each standard, replicate spectra were averaged. In cases where a PC was available from multiple suppliers, the mean spectra from these standards were further averaged, resulting in one representative spectrum per compound. In total, 39 mean spectra (26 flavonoids and 13 phenolic acids) were obtained. In addition, in order to better characterize the richness and relevance of spectral subregions, three progressively narrower spectral ranges were compared: the full range (0.9–20.3 THz, R0), a reduced range (1.5–20.3 THz, R1), and a narrower interval (5–17 THz, R2). These choices are based on the results of the repeatability and reproducibility studies. To avoid edge effects, all pre-processing was applied only after spectral range selection.

**Figure 1:**
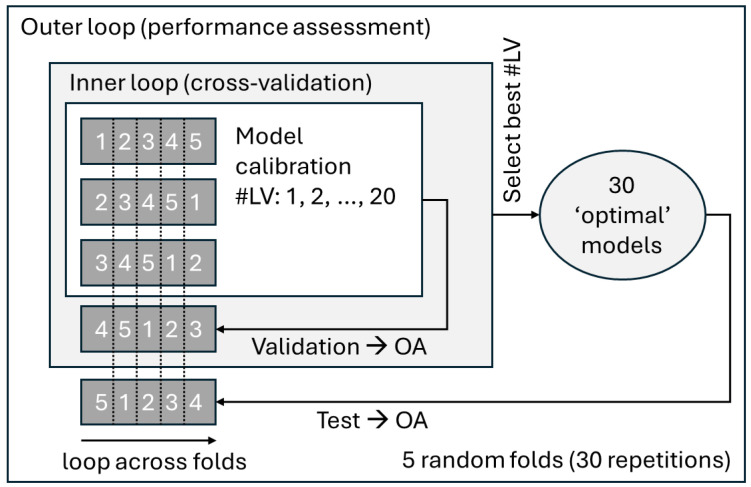
Schematic representation of the double cross-validation procedure.

In order ensure that the results represent well the whole dataset, performance was assessed with double cross-validation (Figure 1). Specifically, the dataset was split into five folds. In the outer loop, one fold was withheld as the test set, while the remaining folds formed the tuning set. Within the tuning set, an inner cross-validation loop was applied: one fold was removed at each turn, models with varying numbers of latent variables (LVs, 1–20) were trained on the remaining folds, and predictions were made on the withheld fold. The number of LVs maximizing overall accuracy (OA) in this inner loop was selected. A final model with this optimal number of LVs was then calibrated on the entire tuning set and evaluated on test set. This nested procedure was repeated 30 times with random fold splits to estimate confidence intervals. OA was used as the main performance metric, which is appropriate given the relatively balanced class distributions in all three case studies. During the process, 30 optimal PLS-DA models with varying numbers of LVs were obtained. For each case study, the most relevant number of LVs was defined as the mode across iterations, and a final model was then calibrated on all observations. For these models, the samples scores and the loadings were calculated and explored.

## 3. Results and Discussion

### 3.1. Data Cleaning

**Figure 2:**
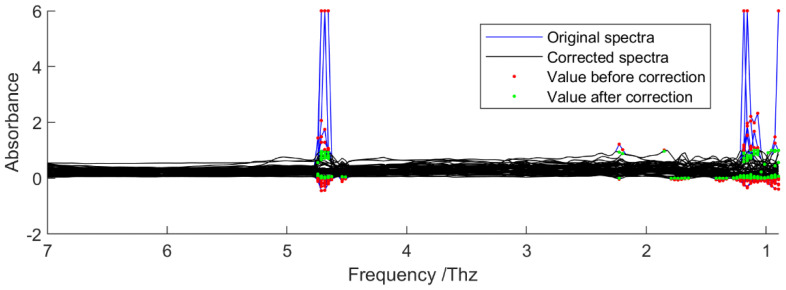
Correction of outlier values in the spectra.

The first observation of the spectra revealed some outlier values at specific frequencies, typically around 1 THz and between 4.5 and 5 THz (Figure 2). All the values of absorbances below 0 or above 1 were replaced by the modified Akima cubic Hermite interpolation using the ‘interp1’ function in MATLAB^®^ Version 23.2.0. Subsequently, any extrapolated values still below 0 or above 1 were set to 0 and 1, respectively. Overall, 55 of the 141 spectra required correction, involving replacement of 205 absorbance values and corresponding to 0.2% of all data points.

### 3.2. Repeatability and Time-Reproducibility

For the repeatability study, multiple spectra of the same sample often exhibited consistent vertical shifts. This is common with powder samples, where subtle variations in particle size and compaction affect light-matter interactions (mainly scattering and absorption), thereby altering the optical path length. However, these effects were largely corrected after pre-processing), resulting in more similar spectra.

**Figure 3:**
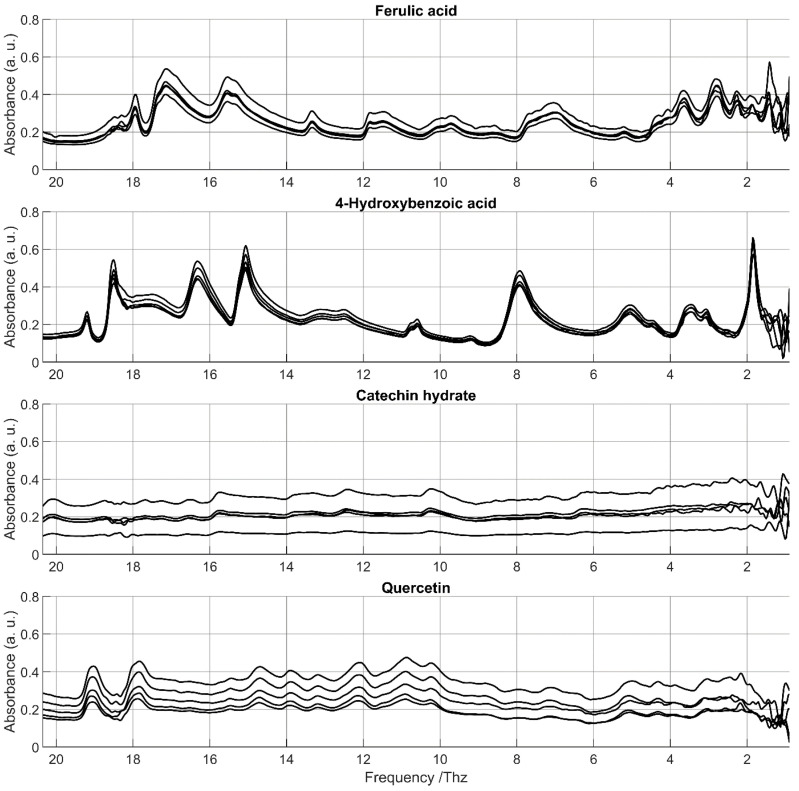
Raw spectra of absorbance of the four PCs selected for the time-reproducibility study, measured at five timepoints separated by one week.

In the reproducibility study, vertical shifts were also apparent in the raw spectra (Figure 3) but were successfully removed by the pre-processing workflow. Except for these vertical shifts, spectra recorded on different dates were very similar. Minor inconsistencies were observed mainly in the spectral range extremes, especially in the low-frequency region.

**Figure 4:**
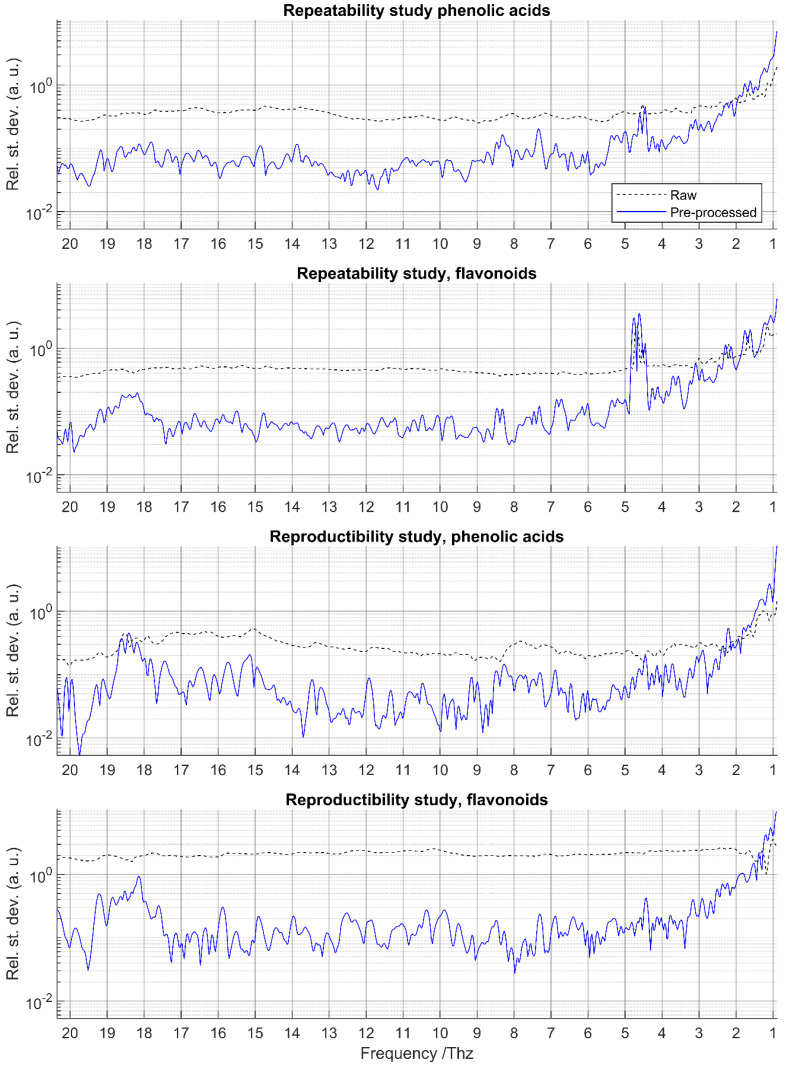
Mean RSD for the raw and pre-processed spectra of phenolic acids and flavonoids. For the repeatability study, classes correspond to repeated measurements of the same sample. For the time-reproducibility study, classes correspond to measurement of the same standard at five times.

The mean relative standard deviations (RSD) of raw and pre-processed spectra for repeatability and reproducibility assessments are shown in Figure 4 for phenolic acids and flavonoids, respectively. In both studies, raw spectra exhibited substantially higher RSDs than pre-processed spectra, mainly due to vertical baseline shifts. After pre-processing, RSD values were consistently low (≈0.1) except in three specific regions.

First, at low frequencies (<4 THz), the mean RSD increased exponentially, exceeding 1.5, suggesting a progressive loss of measurement performance in this range. Second, in the repeatability study of flavonoids, a localized increase in RSD (>0.5) was observed between ~4.5 and 5 THz; this region coincides with the frequencies where absorbance corrections were previously applied (see Section 3.1). Finally, for flavonoids in the repeatability study and for both families in the reproducibility study, elevated RSD values (up to ~0.5) appeared around 18–19 THz.

On the basis of these findings, three progressively narrower frequency ranges (R0, R1, and R2; see Section 2.5) were defined for subsequent predictive classification modeling.

### 3.3. FIR Characterization of Phenolic Compounds

The raw FIR spectra of all phenolic compounds are provided in the Supplementary Material. At first inspection, no clear differences could be distinguished between phenolic acids and flavonoids. Both families displayed spectra with either simple or complex profiles. Unlike in the MIR region, where phenolic acids generally exhibit better-resolved spectra than flavonoids [6], no such distinction was apparent in the FIR range. This lack of obvious differences highlights the need for chemometric approaches to explore whether subtle spectral features can still enable reliable classification. The following sections discuss the main patterns present in the spectra of individual PCs.

#### 3.3.1. Phenolic Acids

Phenolic acids contain a carboxyl group attached to a benzene ring. Two main classes of phenolic acids are distinguished based on their structures: benzoic acid derivatives (hydroxybenzoic acids, C6-C1) and cinnamic acid derivatives (hydroxycinnamic acids, C6-C3) [15]. Samples were classified accordingly. The Supplementary Material shows many spectral differences among phenolic acids.

Within hydroxybenzoic acids, structural complexity varies widely. The simplest acids, 4-hydroxybenzoic acid and salicylic acid, differ only in the hydroxyl group position but display distinct absorption bands. 4-Hydroxybenzoic acid shows bands at approximately 1.8, 7.9, 15.1, 16.3, 18.5, and 19.2 THz, while salicylic acid exhibits bands near 2.1, 8.4, 11.1, 12.8, 13.8, 15.9, 17.0, and 19.7 THz.

Dihydroxybenzoic acids show varying spectral complexity: protocatechuic acid (3,4-dihydroxybenzoic acid) presents fewer bands at 1.33, 7.4, 16.3, and 19.3 THz; gentisic acid (2,5-dihydroxybenzoic acid), despite a similar structure, has a richer profile with numerous bands spanning the entire range. Gallic acid (a trihydroxybenzoic acid) displays a highly informative spectrum with clear bands, notably at approx. 2.1, 2.5, 3.1, 3.6, 3.9, 7.2, 8.8, 9.7, 10.5, 11.1, 15.9, and 16.7 THz. The band at ~2.1 THz has already been revealed using THz-TDS [16].

Vanillic acid has a relatively simple chemical structure, consisting of the common backbone of a carboxyl group attached to a benzene ring, with the addition of a single methoxy group (–O–CH_3_). Its spectrum shows distinct absorption bands across the entire range, with a particularly intense and broad band around 15 THz. By contrast, syringic acid, which is structurally more complex due to the presence of two methoxy groups, displays a much simpler spectral profile. It shows only a few absorption bands, the most prominent being at 3.7 THz. Ellagic acid, a more complex dilactone of hexahydroxydiphenic acid, exhibits a markedly different profile characterized by numerous small absorption bands and one dominant peak around 5 THz.

Among the hydroxycinnamic acids, 2-coumaric acid (2-hydroxycinnamic acid) exhibits a relatively simple and well-resolved spectrum, with distinct absorption bands at approximately 2.1, 5.2, 8.1, 13.8, 14.8, 15.4, 17.1, and 17.7 THz. Although caffeic acid differs from 2-coumaric acid only by the presence of an additional hydroxyl group, its FIR spectrum was markedly different, with absorption bands at other frequencies, including three major bands at ~1.3, 4.5, and 7.2 THz. The first one can be related to the sharp absorption peak observed at 1.36–1.39 THz with THz-TDS [17,18]. Ferulic acid and sinapic acid, which contain one and two methoxy groups, respectively, display spectra with a similar overall pattern up to 12 THz, sharing several apparent bands (at ~2.3, 3.8, 5.2, and 11.9 THz). For these two PCs, the comparison with THz-TDS results [17,18] is not possible, due to the low repeatability over this range in our study, with only two repetitions showing dissimilar spectral patterns. Chlorogenic acid, a chemically more complex phenolic acid (an ester of caffeic acid with quinic acid), shows a much richer profile with numerous small bands across the range. Unlike the other hydroxycinnamic acids, no clear common bands could be identified. Two broad absorption features were observed between 2 and 3 THz and around 18 THz. A band at approx. 1.3 THz that, has been observed with THz-TDS [17], is also visible.

In a previous study in the MIR range, [6] also reported spectral variability among phenolic acids, related in part to the number and position of hydroxyl and methoxy groups. However, the present work reveals even more pronounced spectral differences between structurally similar molecules.

#### 3.3.2. Flavonoids

The flavonoid group encompasses different classes and comprises a large number of molecules. All flavonoids share the general C6–C3–C6 backbone, but varying degrees of oxidation, methoxylation, and glycosylation across the different rings generate extensive structural heterogeneity. Not all classes were investigated in this study, but several representatives were included, with particular emphasis on two major classes, flavanols and flavonols. Molecules from other classes (anthocyanins, dihydrochalcones, flavones, flavanones, and isoflavones) were also analyzed.

Flavanols

Catechin hydrate and epigallocatechin display similar spectra, with only a few intense bands between 1 and 5 THz (at ~1.2, 1.8, and 4.7 THz). Above 5 THz, their profiles are relatively smooth. The band at ~4.7 THz is also observed in epicatechin gallate and epigallocatechin gallate. Despite sharing the same basic structure, epicatechin and epicatechin gallate exhibit highly dissimilar spectra, with only two bands in common (~4.9 and 19.4 THz). Catechin and epigallocatechin gallate show similar profiles with small bands across the full range, but unexpectedly, they do not resemble other flavanols despite their close chemical structures. Catechin also lack clear common bands with epicatechin, which differs only in configuration (catechin: trans isomer; epicatechin: cis isomer). Overall, the flavanols studied here produce markedly dissimilar FIR profiles, suggesting that stereochemistry and the presence of additional groups (e.g., water or gallic acid) strongly influence spectral features.

Flavonols

Most flavonols show informative spectra with many bands across the range. Quercetin and quercetin dihydrate present nearly identical profiles above 2 THz, indicating that co-crystallized water does not significantly modify intermolecular vibrations through hydrogen bonding. Below 2 THz, interpretation is more complex due to reduced repeatability. Major bands are found at ~2.1, 4.2, 4.6, 11.4, 12.2, 17.3, 18, and 19 THz, with the band at ~2.1 THz consistent with other studies using THz-TDS [4,13]. By contrast, quercetin-3-glucoside exhibits a much simpler profile, with only one clear band at 4.5 THz, suggesting that glycosylation reduces spectral richness. Quercetin anhydrous has a profile resembling quercetin and quercetin dihydrate, but with additional bands. In agreement, [4] reported an absorption peak at 2.16 THz for quercetin using THz-TDS (0.2–2.5 THz), although higher-frequency bands were not observed in that narrower range. Isorhamnetin, which differs from quercetin by one methoxyl group, shares only a few bands with this latter (~11.4 and 19 THz) but presents numerous additional ones. Kaempferol and myricetin, chemically very close to quercetin (differing only by one or two hydroxyl groups), also displays distinct profiles, with only a single common band at ~17.5 THz (likely corresponding to the quercetin band at 17.3 THz). The band observed at approx. 2.1 THz for these two PCs with THz-TDS [13] could not be observed due to low repeatability in our spectra in the corresponding range. Kaempferol also shares the 19 THz band observed in quercetin. Absorbance levels are generally lower for kaempferol. Rutin and rutin hydrate show highly similar spectra, but distinct from the other flavonols: both contained many small bands across the range and one predominant band at ~4.5 THz, likely due to their glycosylated structure (quercetin bound to rutinose).

Other flavonoids

Anthocyanins generally produce poor spectra; only kuromanin chloride exhibits a few identifiable bands (~13, 17.6, and 17.8 THz). In contrast, compounds from other classes display more informative profiles. Phlorizin and phloretin (dihydrochalcones) have distinct spectra despite their close relationship (phlorizin being the glucoside of phloretin), with only two common bands (~12.5 and 14.3 THz), again highlighting the strong impact of glycosylation. Luteolin (flavone) and eriodictyol (flavanone) differ only by a double bond in the C-ring (2,3-position), yet their spectra are distinct; luteolin spectrum is more informative, with only two bands in common (~14.6 and 16.5 THz). Interestingly, a band at 10 THz is shared between luteolin and phloretin. Daidzein (isoflavone) displays numerous bands, including low-frequency ones at 1.24 and 1.75 THz also reported by [4]. Other bands described in that study (2.04 and 2.39 THz) are less clear here, while additional features at ~2.7, 3.6, 4.5, 6.4, 14.6, 15.8, and 16.3 THz are observed. Despite limited repeatability at this range, genistein (isoflavone) shows bands at approx. 1.74 and 2.01 THz, as observed by THz-TDS [4]. It also shows many additional bands across the range.

Overall observations

Across all families and classes of phenolic compounds, spectra reveal highly distinct molecular fingerprints. No general trend could be identified, and common bands are rare, even between closely related molecules. These findings confirm and extend the observations of [4], who concluded that flavonoids, despite their structural similarities, exhibit markedly different absorption peaks in the THz region. This reflects the complex vibrational modes of biomolecules, which involve intramolecular vibrations, rotational modes, and weak intermolecular interactions such as hydrogen bonds and van der Waals forces.

### 3.4. Predictive Classification Modeling

As introduced earlier, a double cross-validation procedure was applied using PLS-DA with five randomly selected folds and thirty repetitions. The overall accuracies (OAs) for the three case studies (CS1, CS2, and CS3) and the three frequency ranges (R0, R1, and R2) are reported in Table 3.

For CS1 (flavonoids vs. phenolic acids), the average OA across repetitions was always ≥0.964. Both the average and minimal OA values indicate that ranges R1 and R2 performed best, whereas inclusion of the lowest frequencies (<1.5 THz) reduced robustness. This result agrees with the low repeatability and reproducibility previously observed in the low-frequency region.

**Figure 5:**
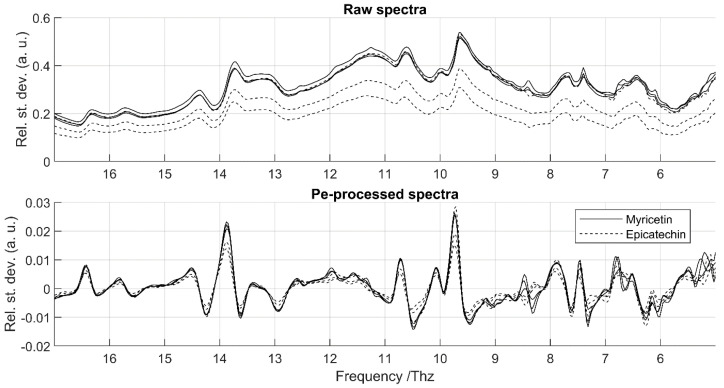
Raw and pre-processed spectra of myricetin and epicatechin highlighting subtle spectral differences. Although averaged spectra were used for calibration, all repetitions are displayed to illustrate variability. The figure is restricted to R2, where the most relevant features are located.

For CS2 (flavonols vs. non-flavonols), the average OA was consistently above 0.972. The highest performance (0.995) was obtained with R2, but accuracy decreased with the broader ranges R0 and R1. This suggests that frequencies below 5 THz and above 17 THz negatively affected classification, again consistent with repeatability issues observed for flavonoids between 4.5 and 5 THz and 18–19 THz. At the compound level, the rate of correct classification was always ≥0.9, except for quercetin-3-β-D-glucoside with R0 (0.767) and epicatechin with R1 (0.767). The poor classification of quercetin-3-β-D-glucoside is likely due to its flat and weakly characteristic spectrum, while for epicatechin it reflects the strong spectral similarity with myricetin (Figure 5), despite their belonging to different classes.

For CS3 (hydroxycinnamic acids vs. hydroxybenzoic acids), the average OA reached 0.974 with R0 but dropped below 0.9 with R1 and R2. This indicates that, despite repeatability issues, the lowest frequency region (<1.5 THz) carries useful discriminatory information for distinguishing between these two classes of phenolic acids.

**Figure 6:**
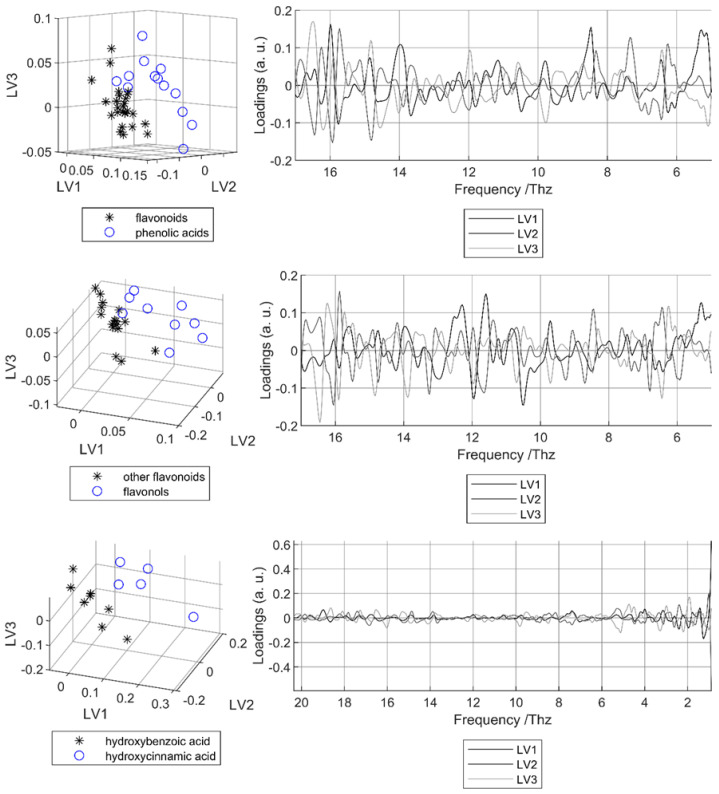
Scatter plots of scores for the three first LVs (**left column**) and plots of the corresponding loadings (**right column**) for the case studies CS1 (**top**), CS2 (**mid position**) and CS3 (**bottom**). For the PLS-DA model, the spectral range with the highest average OA in cross-validation was used.

The sample scores and the loadings of the final models are represented in Figure 6. For all three case studies, the scores of the first three LVs show that the two classes can be separated at least relatively well in a linear way (left column). The associated loadings do not emphasize a single spectral region but instead display multiple minima and maxima across the whole range (right column). These correspond to the positions of absorption bands observed in the spectra of several PCs within each group. This indicates that there are no strong or systematic spectral features uniquely characterizing specific PC classes or families. Instead, discrimination relies on combinations of multiple spectral features arising from the individual PCs within each group, reflecting the inherent structural diversity of phenolic compounds. This also suggests that discriminant information is distributed across the spectrum, though not all regions are necessarily required for class separation. Certain areas appear more informative: in CS1, higher loadings amplitudes are found around 5–6 THz, 8–9 THz, and 13–17 THz; in CS2, the first LV alone explains 61% of class variability, with strongest amplitudes between 5 and 6 THz and 10–13 THz; and in CS3, the most discriminant region lies between 0.9 and 3 THz, despite the lower repeatability observed there.

## 4. Conclusions

This study explored the potential of FIR spectroscopy as an effective, sensitive, and non-destructive approach for identifying and discriminating PCs. To our knowledge, this work presents the first FIR spectra of a broad set of PCs, revealing that band profiles vary widely: some molecules displayed complex signatures, while others with equally complex chemical structures surprisingly showed simpler ones. Thus, chemical structure complexity does not necessarily predict FIR profile richness, and structural similarities between molecules do not guarantee spectral resemblance. The repeatability and time-reproducibility of the pre-processed spectra were generally good across the frequency range, though slightly higher variability was noted at low frequencies (<4 THz), in the 4.5–5 THz interval, and around 18–19 THz.

Most phenolic compounds prove to be highly active in the FIR range, and our results indicate that no unique spectral zone characterizes specific families or classes of PCs. Instead, discrimination results from the combination of several spectral features reflecting the structural diversity of these molecules. Although discriminant information seems to be spread throughout the spectrum, the results indicated that certain frequency ranges appear to be more informative. These findings may be compared to those obtained using MIR and Raman spectroscopy where differentiation between families and/or groups were also considered. In the mid-infrared region, numerous absorption bands were also reported by [6]. However, they could identify two spectral regions as particularly relevant for differentiating between phenolic compound families (phenolic acids and flavonoids) even though clearly family-specific regions were difficult to pinpoint. A similar set of PC was also characterized by Raman spectroscopy by [14]. Bands have been identified and differentiated within and between groups and several scattering intensities were identified to be responsible for differentiating 100% of PC families, classes and subclasses.

Despite this variability, classification models confirmed that the full spectral range was important for discriminating phenolic acids, while flavonoids were best classified with a reduced frequency window, excluding the range extremes. Predictive modeling achieved moderate to nearly perfect accuracy across case studies, with robust performance estimates ensured by double cross-validation despite the limited dataset.

Overall, these results demonstrate the capacity of FIR spectroscopy to discriminate between families and classes of PCs, highlighting its potential as a complementary tool to Raman and conventional MIR-FTIR spectroscopy for the characterization and discrimination of bioactive phenolics.

Terahertz (THz) radiation used in the THz spectroscopy has the properties of both microwave and infrared and can penetrate and interact with many commonly used materials. THz spectroscopy has been investigated as a potential technique to analyze and quantify bioactive compounds in several studies. Recently, [19] demonstrated the effectiveness of Terahertz spectroscopy in the pharmacological quantification of bioactive compounds. In the study of [13], THz spectroscopy was used in combination with chemometrics for the qualitative and quantitative analysis of three flavonols with similar chemical structures (myricetin, quercetin, and kaempferol). They demonstrated the power of this technique for the identification of these compounds and the quantitative determination of their concentrations. The study of [20] addressed the detection of both flavonoids, hesperidin and naringin, by using terahertz spectroscopy in orange peel extracts. In addition to THz spectroscopy, the use THz imaging has been shown to have great potential as an emerging nondestructive tool for food inspection. The THz technique is a great tool for characterization of many components such as carbohydrates, amino acids, fatty acids, and vitamins. A complete review has been carried out by [21].

This study on pure standards represents a first step in evaluating the potential of THz spectroscopy for characterizing phenolic compounds (PCs) in real food products. Moving toward more complex matrices will, however, present challenges. The first is the limited sensitivity of FTIR spectroscopy compared with traditional analytical methods, which may reduce its efficiency for detecting trace compounds. The second is the strong absorption of water in the FIR and THz ranges, which can interfere with the spectral signals of PCs and other components, particularly in fruits, vegetables, or beverages.

To address these limitations, a gradual approach seems appropriate. The next step should involve the characterization of dry mixtures of pure PCs, possibly combined with weakly absorbing excipients. A strong experimental design should be implemented, involving gradual concentration of PCs, pure or mixed together, and different excipients and degrees of humidity. Both detection and quantification should be evaluated. Subsequently, predictive models could be validated on commercially available food supplements enriched with PCs. The insights gained from these studies would help identify additional food products in which efficient characterization of PCs can be achieved.

## Figures and Tables

**Table 1:** Phenolic compounds analyzed in this study, grouped by family and class. Suppliers are listed in the footnote.

Family	Class	Phenolic Compound
Phenolic acid	Hydroxybenzoic acid	gentisic acid ^1,2,3^; ellagic acid ^1,2,3^; syringic acid ^1,2,3^; vanillic acid ^1,2,3^, gallic acid ^1,2^; salicylic acid ^2^; 4-hydroxybenzoic acid ^2,3^; protocatechuic acid ^2,3^;
	Hydroxycinnamic acid	caffeic acid ^1,2,3^; chlorogenic acid ^1,2^; sinapic acid ^2^; ferulic acid ^2^; 2-coumaric acid ^2,3^;
Flavonoid	Flavanol	catechin ^3^; catechin hydrate ^1^; epicatechin ^3^; epigallocatechin ^3^; epicatechin gallate ^3^; epigallocatechin gallate ^3^;
	Flavonol	quercetin anhydrous ^2^; quercetin dihydrate ^1^; quercetin-3-b-D-glucoside ^2^; quercetin ^1^; rutin hydrate ^2^; rutin ^3^; kaempferol ^2^; myricetin ^2^; isorhamnetin ^3^;
	Anthocyanin	cyanidin chloride ^3^; keracyanin chloride ^3^; kuromanin chloride ^3^; myrtillin chloride ^3^;
	Dihydrochalcone	phloretin ^2^; phlorizin ^2^;
	Flavone	luteolin ^1,3^;
	Flavanone	eriodictyol ^2^; bavachinin ^3^;
	Isoflavone	genistein ^3^; daidzein ^3^;

^1^ VWR; ^2^ Sigma-Aldrich; ^3^ Extrasynthese.

**Table 2:** Phenolic compounds used in the time-reproducibility study.

Phenolic Compound	Family	Class	Supplier
Ferulic acid	Phenolic acid	Hydroxycinnamic acid	Sigma-Aldrich
4-Hydroxybenzoic acid	Phenolic acid	Hydroxybenzoic acid	Extrasynthese
Catechin hydrate	Flavonoid	Flavanol	VWR
Quercetin	Flavonoid	Flavonol	Sigma-Aldrich

**Table 3:** Average overall accuracy (OA) achieved in discriminating classes for three case studies: CS1 (flavonoids vs. phenolic acids), CS2 (flavonols vs. other flavonoids), and CS3 (hydroxycinnamic acids vs. hydroxybenzoic acids). Results are shown for three spectral frequency ranges: R0 (full range), R1 (1.5–20.3 THz), and R2 (5–17 THz). Values in parentheses indicate the minimum and maximum OA across thirty repetitions.

	R0	R1	R2
**CS1** **CS2** **CS3**	0.964 (0.692–1.000)	0.985 (0.872–1.000)	0.987 (0.872–1.000)
	0.972 (0.769–1.000)	0.979 (0.923–1.000)	0.995 (0.962–1.000)
	0.974 (0.692–1.000)	0.874 (0.692–1.000)	0.892 (0.538–1.000)

## Data Availability

To facilitate collaborations and ensure proper use, the data underlying this study are available from the corresponding author upon reasonable request.

## Supplementary Materials

The following supporting information can be downloaded at: https://www.mdpi.com/article/10.3390/foods14213737/s1, spectra of standards of phenolic compounds (PCs), including different suppliers and repetitions, and comparisons between mean spectra of each PC.

## Abbreviations

The following abbreviations are used in this manuscript:

FTIR	Fourier transform infrared spectroscopy
PC	Phenolic compound
THz	Terahertz
HPLC	High-performance liquid chromatography
GC	Gas chromatography
GC–MS	Gas chromatography–mass spectrometry
NIR	Near-infrared
MIR	Mid infrared
FIR	Far infrared
SNR	Signal-to-noise ratio
PLS-DA	Partial least squares discriminant analysis
LV	Latent variable
CS#	Case study #
R#	Range #
