# Supplementary figures and images for: Characterization and Discrimination of Pure Standards of Phenolic Compounds Using FTIR Spectroscopy in the Terahertz Range
Source: Foods. 2025 Oct 31;14(21):3737. doi: 10.3390/foods14213737 (PMC12607551; doi:10.3390/foods14213737)

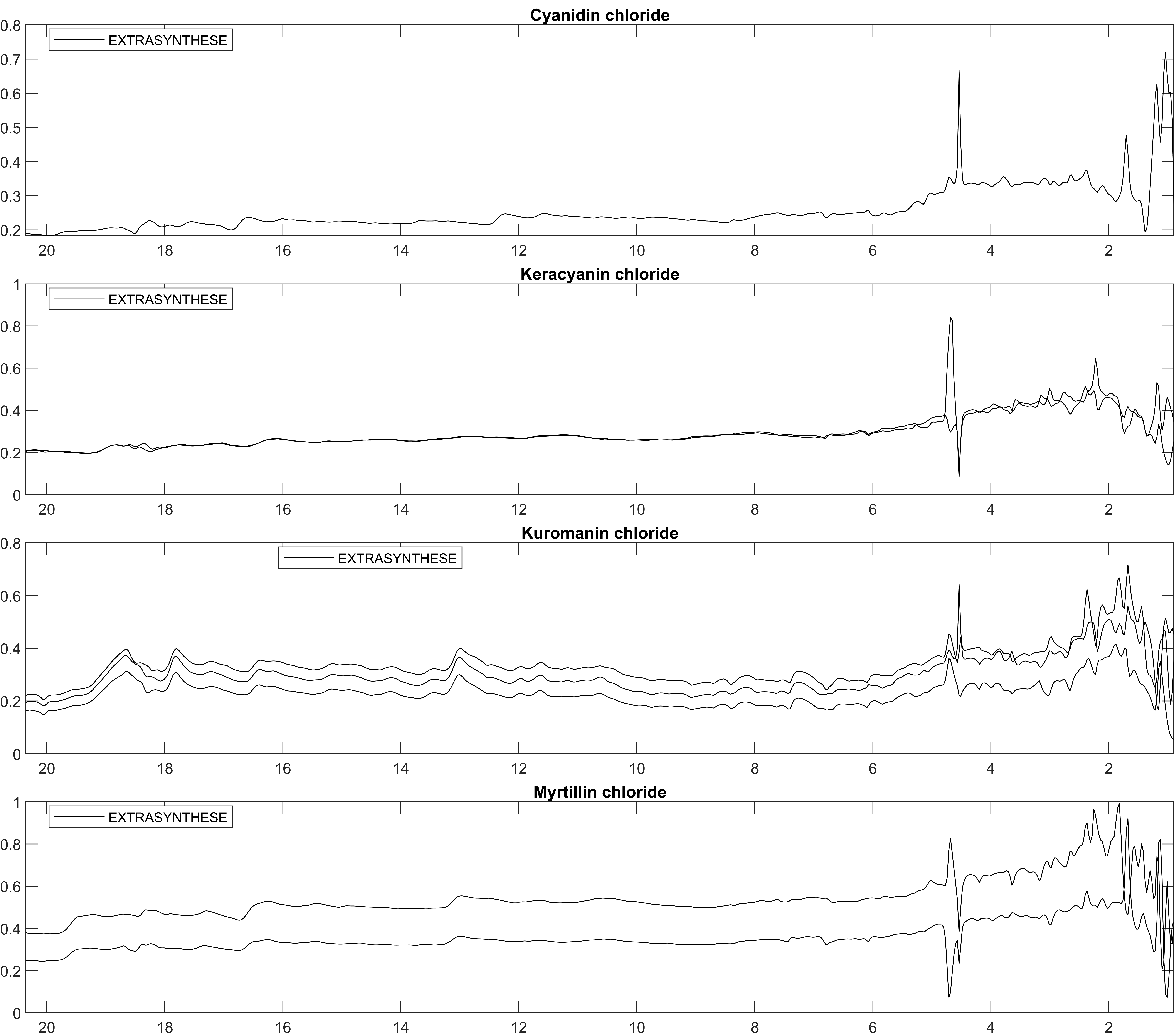

Supplement: Supplementary file 1 [file foods-14-03737-s001.zip › Spectra_standards_Anthocyanin.png]

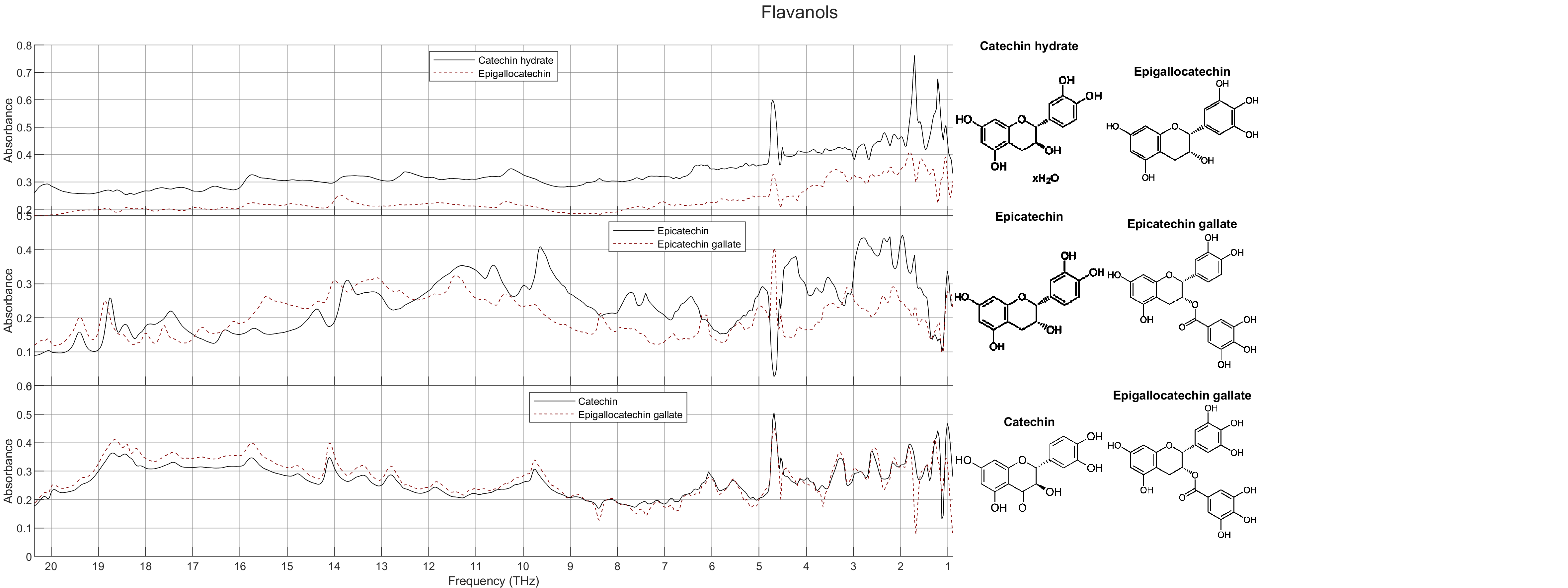

Supplement: Supplementary file 1 [file foods-14-03737-s001.zip › Comparison_spectra_Flavanols.png]

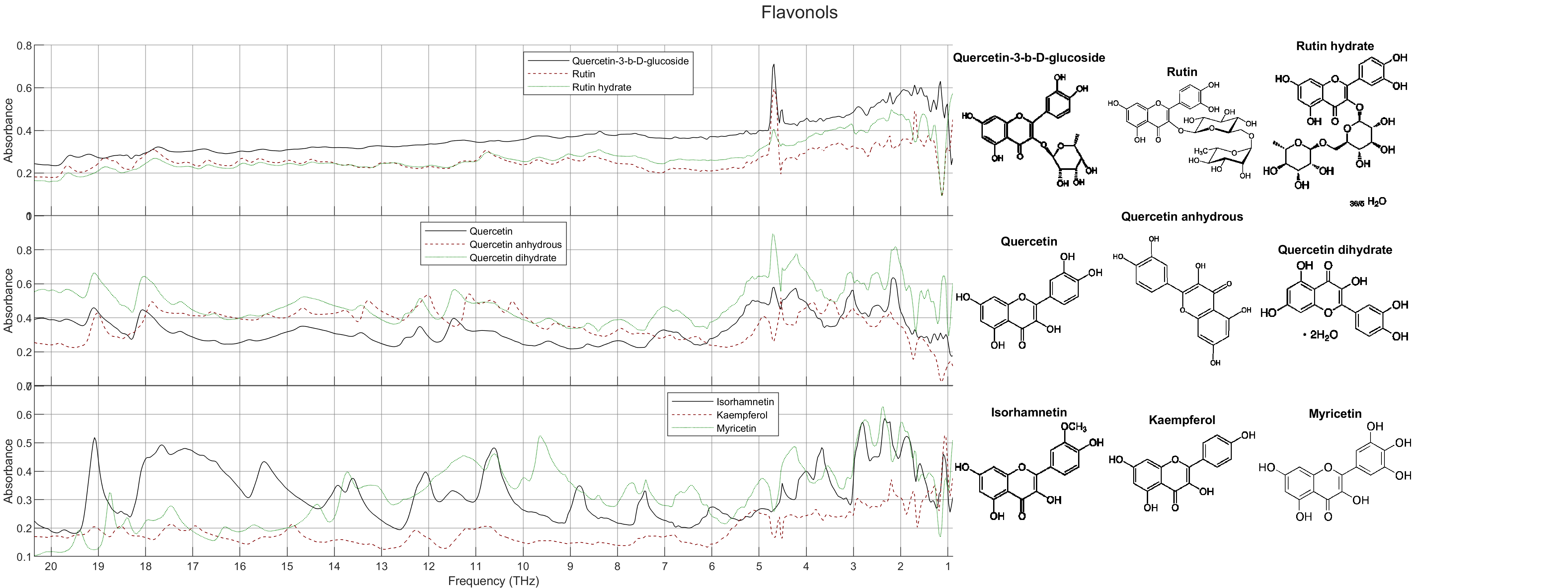

Supplement: Supplementary file 1 [file foods-14-03737-s001.zip › Comparison_spectra_Flavonols.png]

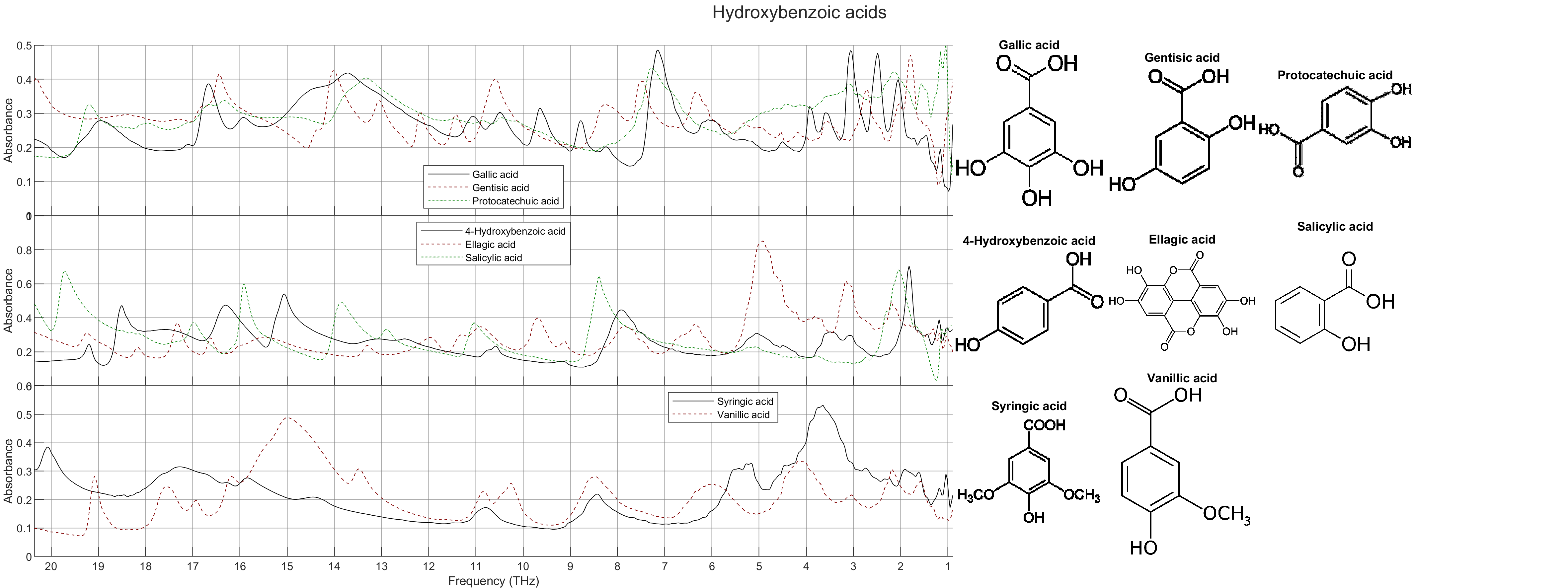

Supplement: Supplementary file 1 [file foods-14-03737-s001.zip › Comparison_spectra_Hydroxybenzoic.png]

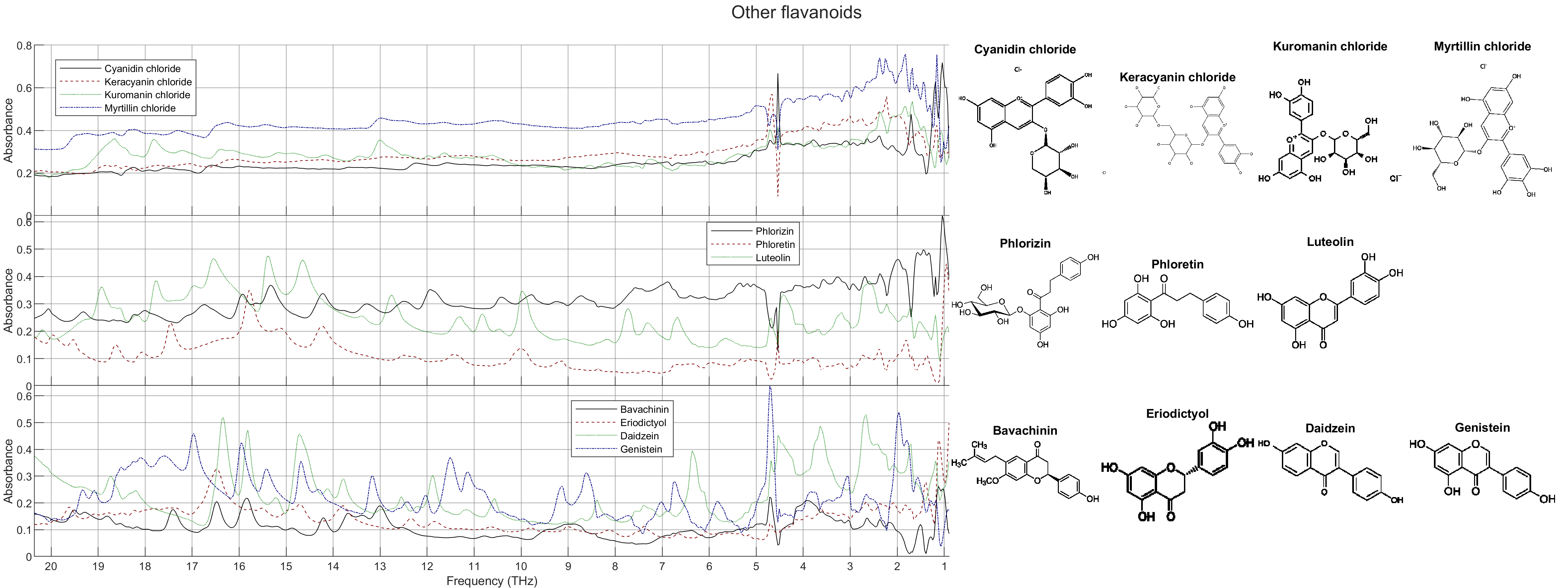

Supplement: Supplementary file 1 [file foods-14-03737-s001.zip › Comparison_spectra_Other.png]

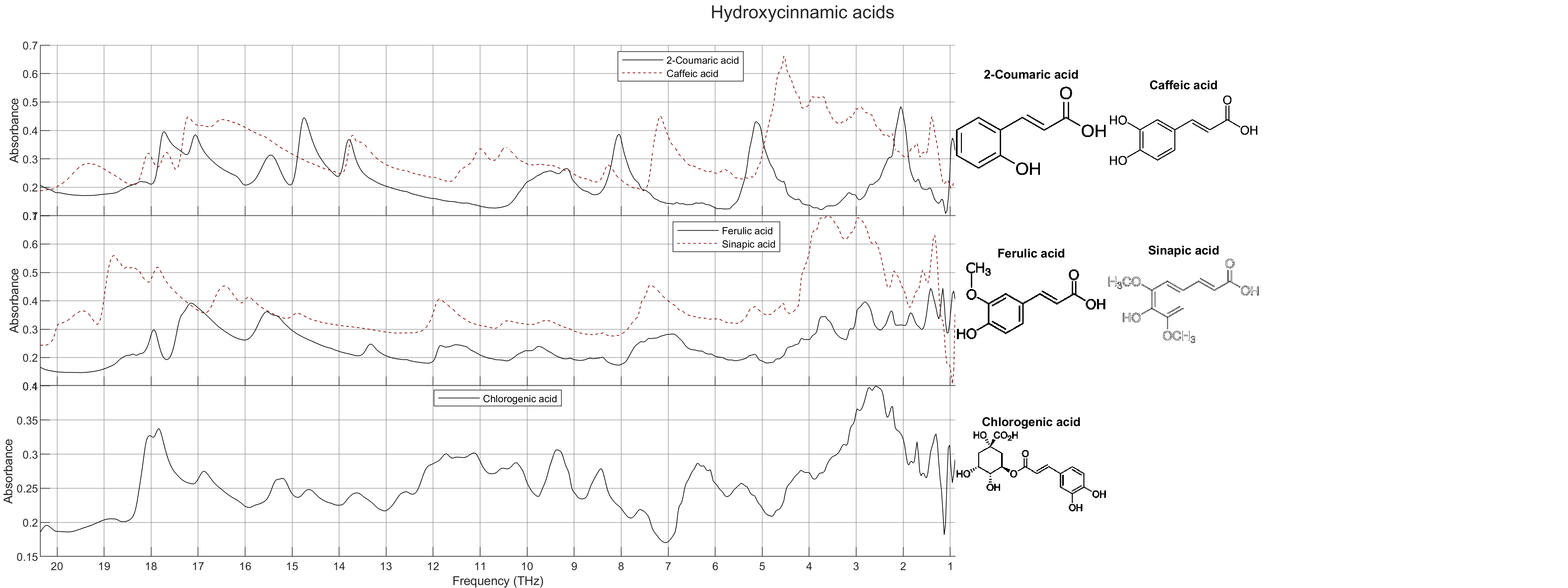

Supplement: Supplementary file 1 [file foods-14-03737-s001.zip › Comparison_spectra_Hydroxycinnamic_acids.png]

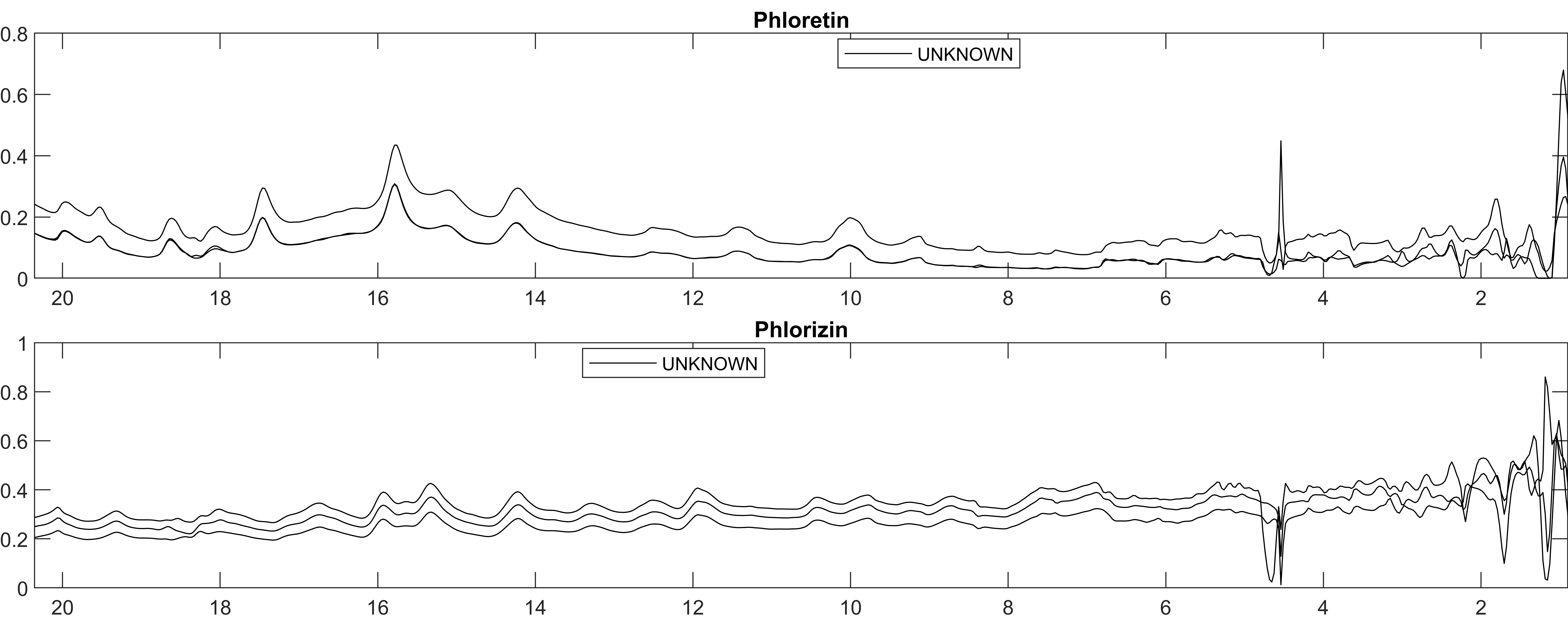

Supplement: Supplementary file 1 [file foods-14-03737-s001.zip › Spectra_standards_Dihydrochalcone.png]

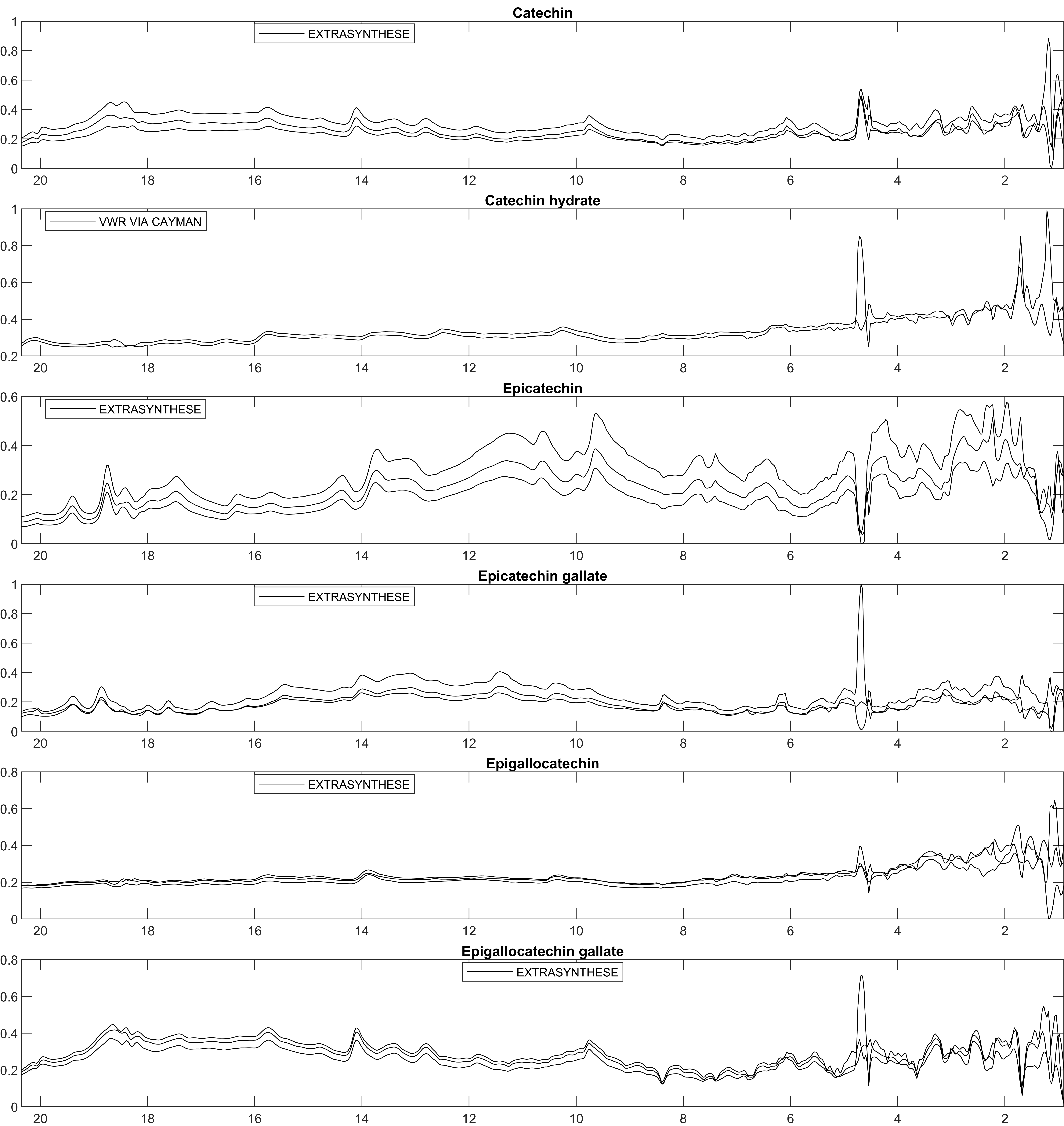

Supplement: Supplementary file 1 [file foods-14-03737-s001.zip › Spectra_standards_Flavanol.png]

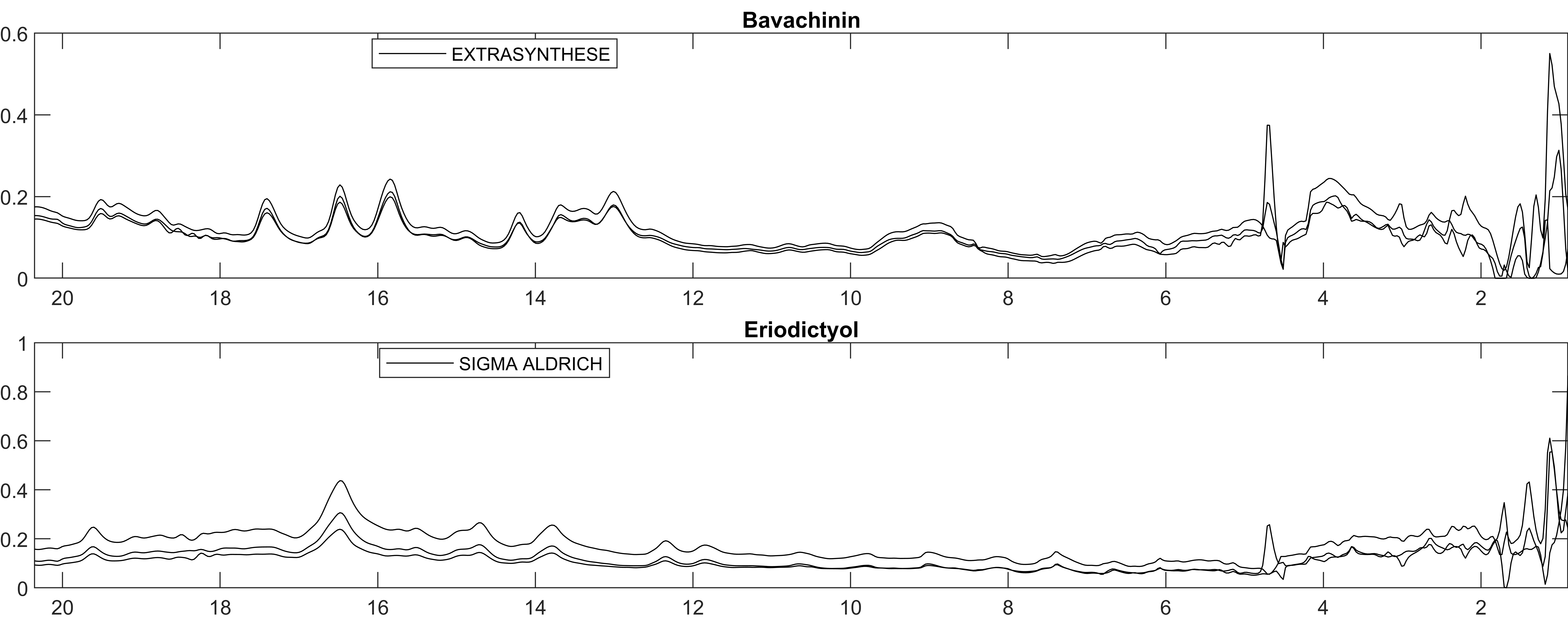

Supplement: Supplementary file 1 [file foods-14-03737-s001.zip › Spectra_standards_Flavanone.png]

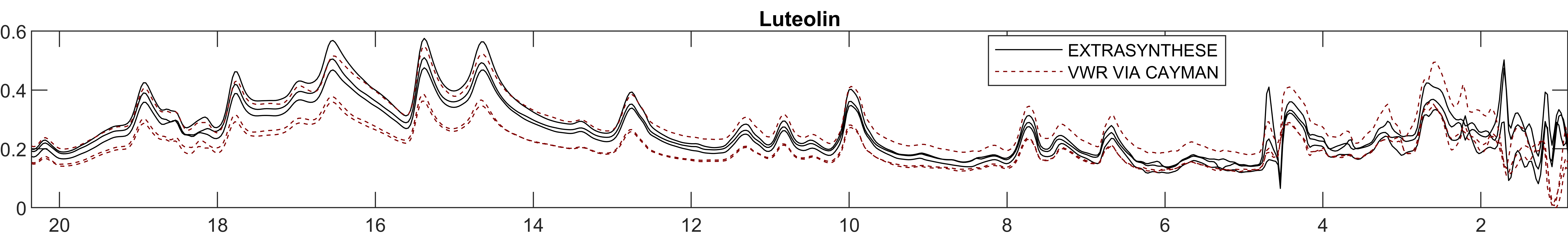

Supplement: Supplementary file 1 [file foods-14-03737-s001.zip › Spectra_standards_Flavone.png]

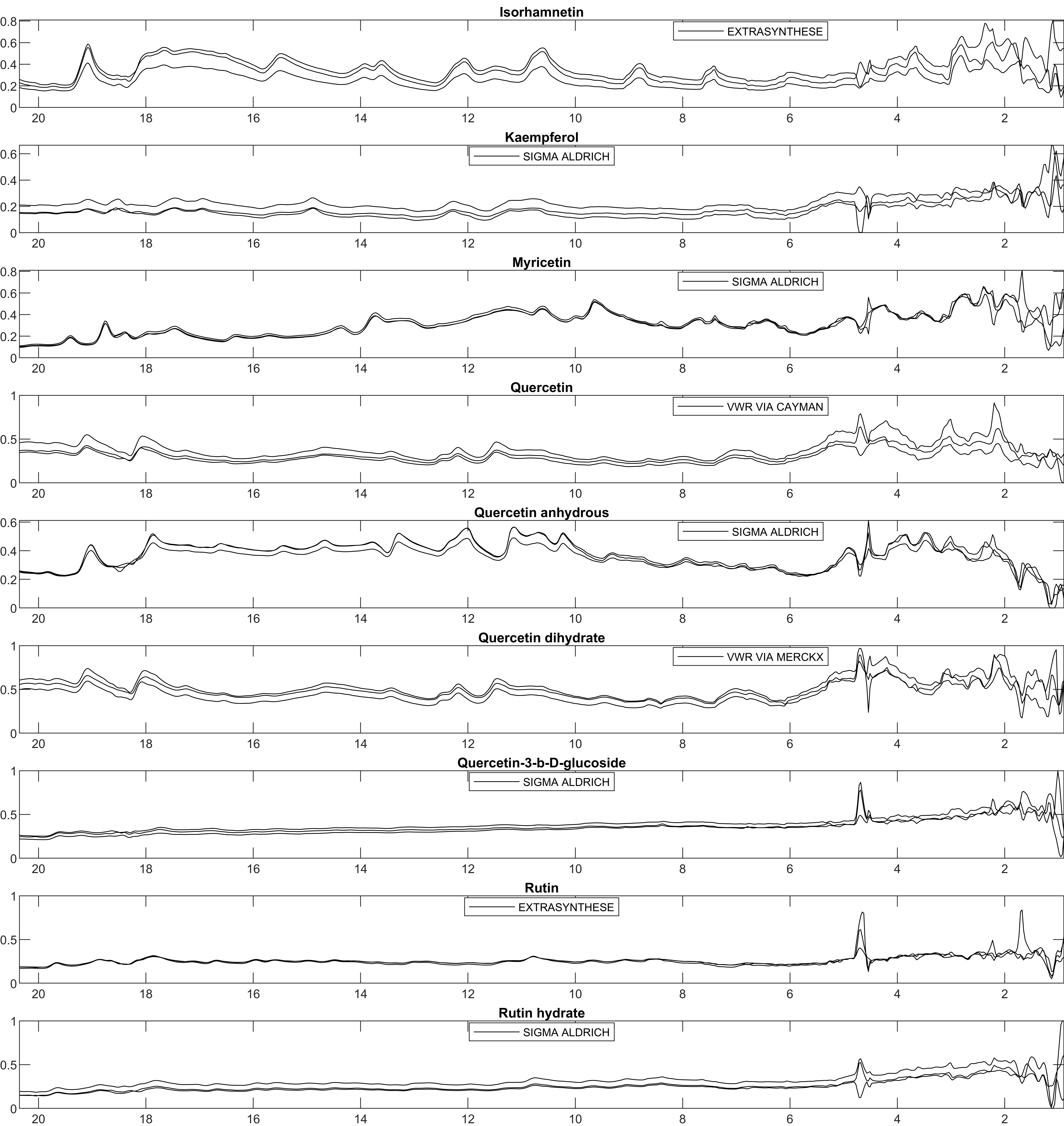

Supplement: Supplementary file 1 [file foods-14-03737-s001.zip › Spectra_standards_Flavonol.png]

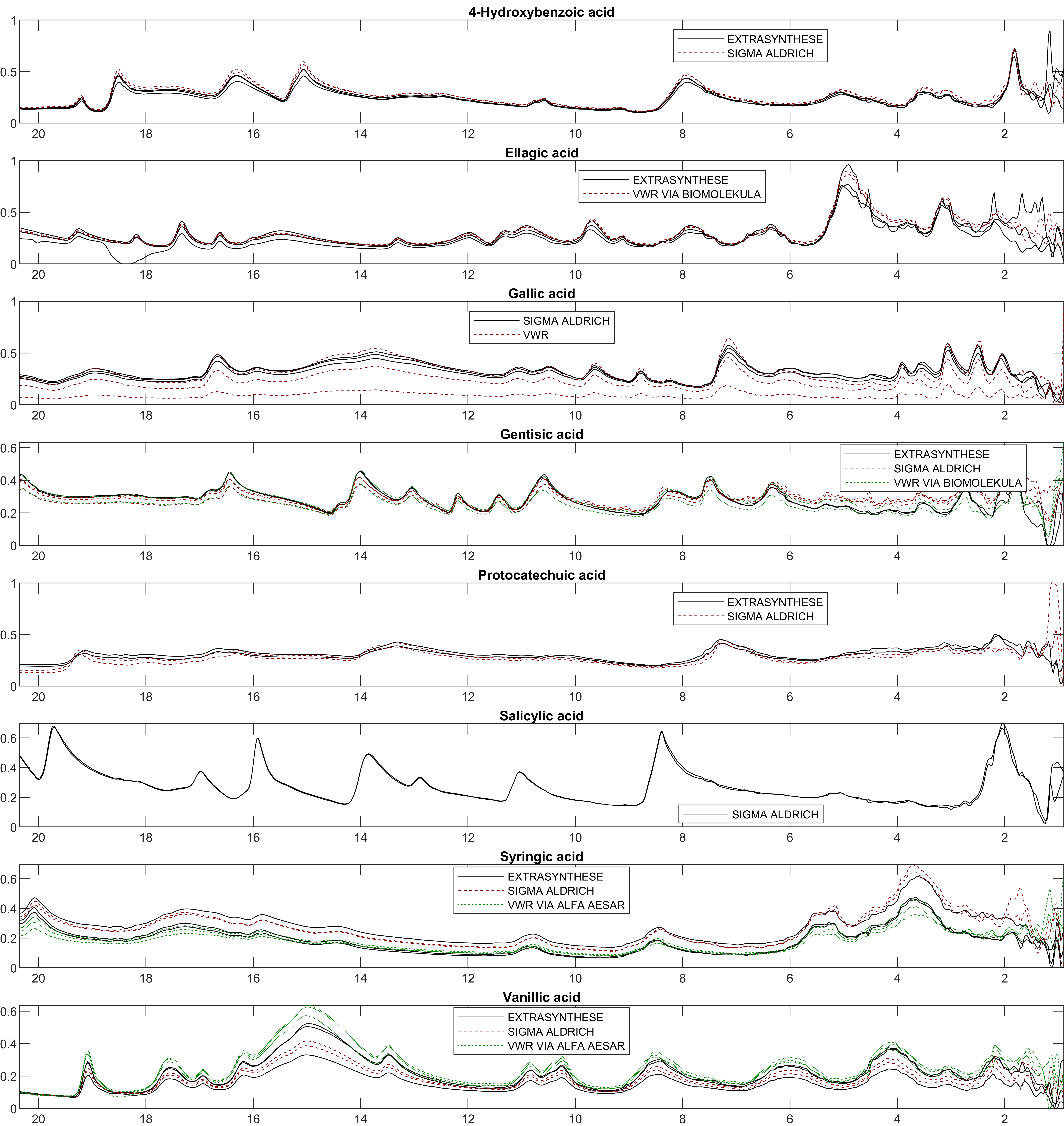

Supplement: Supplementary file 1 [file foods-14-03737-s001.zip › Spectra_standards_Hydroxybenzoic_acids.png]

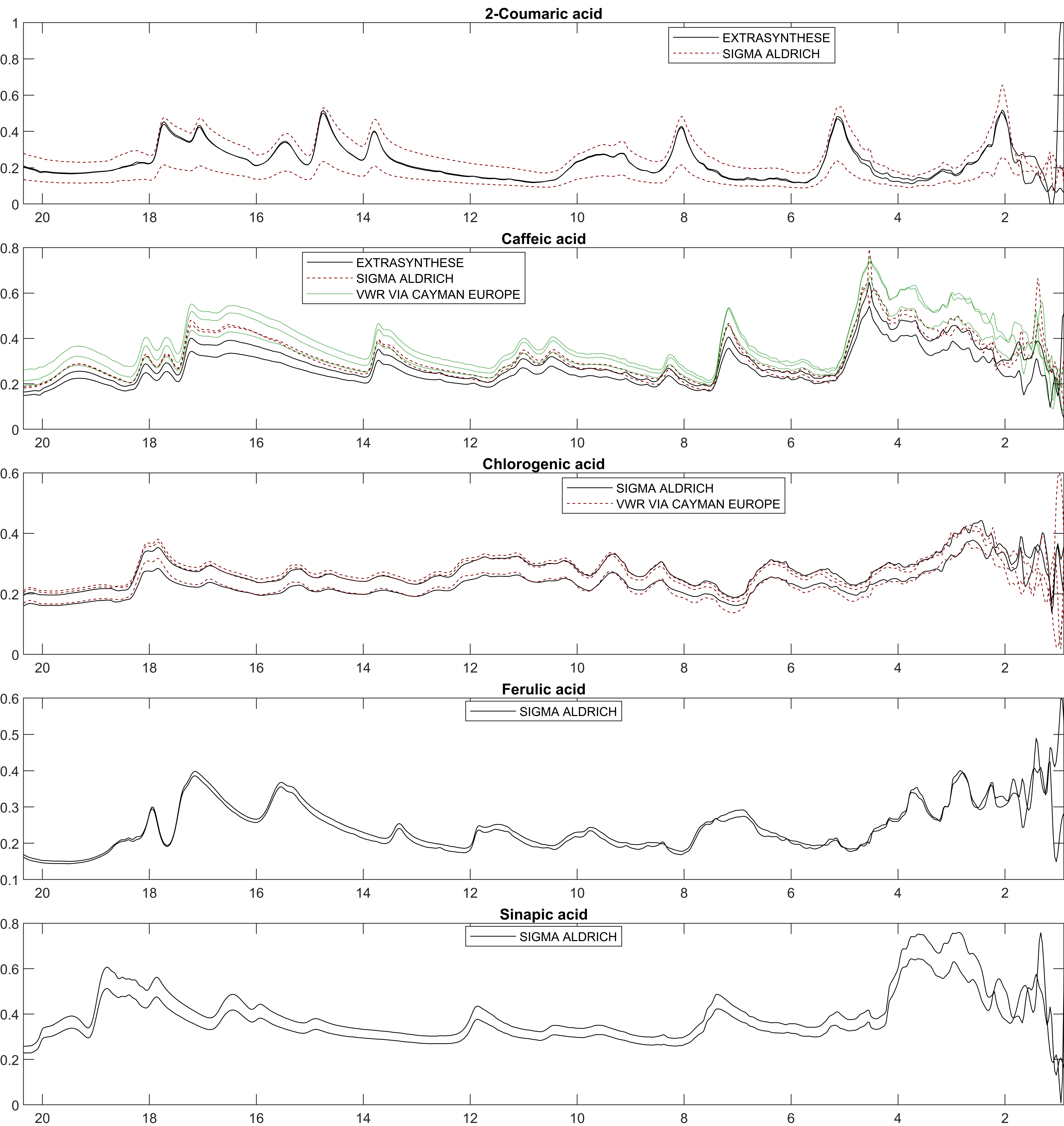

Supplement: Supplementary file 1 [file foods-14-03737-s001.zip › Spectra_standards_Hydroxycinnamic_acid.png]

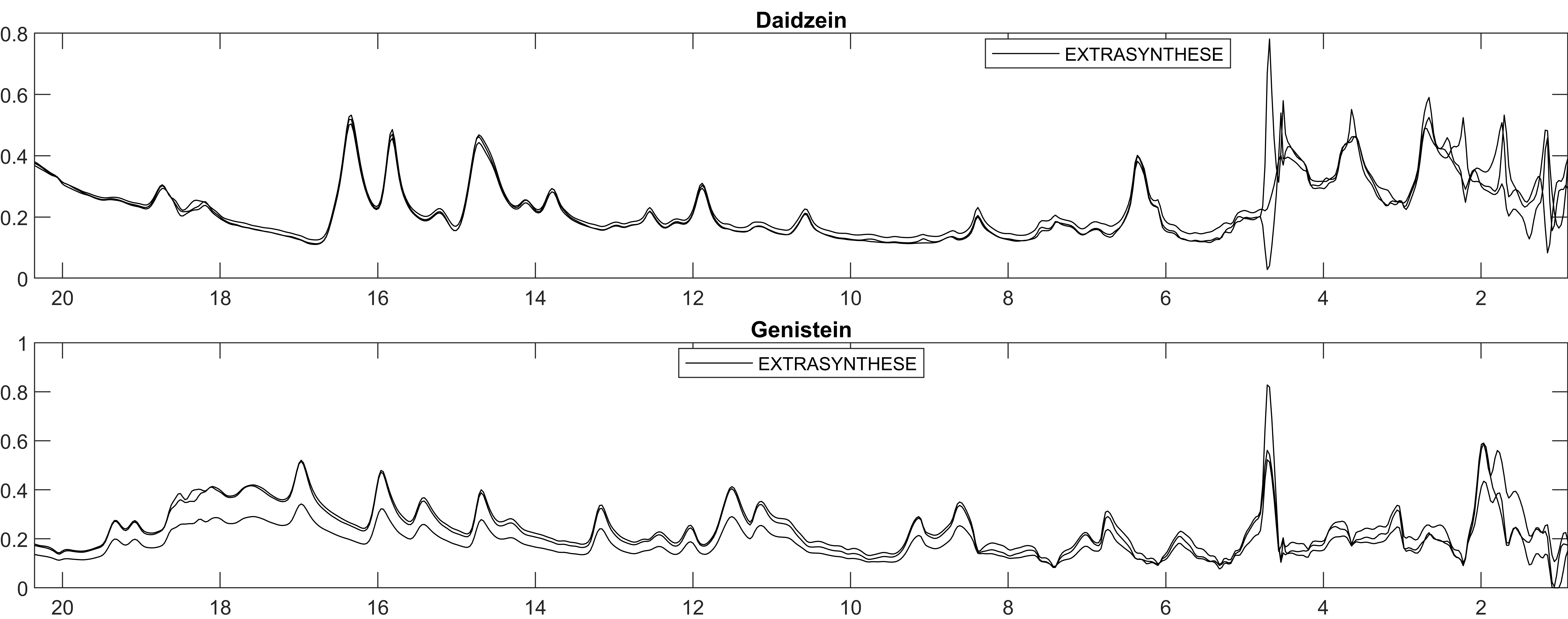

Supplement: Supplementary file 1 [file foods-14-03737-s001.zip › Spectra_standards_Isoflavone.png]
